# Identification and Characterization of the Lamprey High-Mobility Group Box 1 Gene

**DOI:** 10.1371/journal.pone.0035755

**Published:** 2012-04-26

**Authors:** Yue Pang, Rong Xiao, Xin Liu, Qingwei Li

**Affiliations:** 1 Institute of Marine Genomics and Proteomics, Liaoning Normal University, Dalian, China; 2 College of Life Science and Technology, Dalian University, Dalian, China; National Institute on Aging, United States of America

## Abstract

High-mobility group box 1 (HMGB1), a highly conserved DNA-binding protein, plays an important role in maintaining nucleosome structures, transcription, and inflammation. We identified a homolog of HMGB1 in the Japanese lamprey (*Lampetra japonica*). The *Lampetra japonica* HMGB1 gene (Lj-HMGB1) has over 70% sequence identity with its homologs in jawed vertebrates. Despite the reasonably high sequence identity with other HMGB1 proteins, Lj-HMGB1 did not group together with these proteins in a phylogenetic analysis. We examined Lj-HMGB1 expression in lymphocyte-like cells, and the kidneys, heart, gills, and intestines of lampreys before and after the animals were challenged with lipopolysaccharide (LPS) and concanavalin A (ConA). Lj-HMGB1 was initially expressed at a higher level in the heart, but after treatment with LPS and ConA only the gills demonstrated a significant up-regulation of expression. The recombinant Lj-HMGB1 (rLj-HMGB1) protein bound double-stranded DNA and induced the proliferation of human adenocarcinoma cells to a similar extent as human HMGB1. We further revealed that Lj-HMGB1 was able to induce the production of tumor necrosis factor-α (TNF-α), a pro-inflammatory mediator, in activated human acute monocytic leukemia cells. These results suggest that lampreys use HMGB1 to activate their innate immunity for the purpose of pathogen defense.

## Introduction

High-mobility group B1 (HMGB1) was first identified as a non-histone chromatin-associated factor that plays important roles in gene transcription [1]–[3]. It is a highly conserved protein in vertebrate species [3]–[5] and is ubiquitously distributed in the nuclei and cytoplasm of nearly all cell types [6]. Nuclear HMGB1 binds to DNA, stabilizes nucleosome formation, assists in DNA mismatch repair, and interacts with transcription factors and other proteins [7]. Extracellular HMGB1 can induce a variety of cellular responses, including the expression of proinflammatory mediators, such as tumor necrosis factor-α (TNF-α) [8]–[10], tumor metastasis [11]–[13], and the maturation of dendritic cells [14]–[17]. Furthermore, HMGB1 is actively released via the stimulation of the innate immune system by exogenous, pathogen-derived molecules and is passively released after cell injury in the absence of invasion [17]. Therefore, HMGB1 plays an essential role in the inflammatory cascade by alerting the immune system to tissue damage and impending danger and by subsequently stimulating the immune response to protect the organism [18], [19]. In addition, HMGB1 also participates in other physiological processes, such as cell proliferation, differentiation and apoptosis [20], [21].

Lampreys are one of the most ancient vertebrates alive today, making them an ideal animal model for studying vertebrate evolution, embryo development, and the origin of adaptive immunity [22], [23]. Jawless vertebrates (lampreys and hagfishes) use variable lymphocyte receptors (VLR) as counterparts of the immunoglobulin-based receptors that jawed vertebrates use for antigen recognition [24], [25], and the type of VLR expressed is specific to the lymphocyte lineage: T-like lymphocytes and B-like lymphocytes [26]. Studies have focused on lamprey proteins that have roles in important physiological functions, such as anesthesia, anticoagulation and vasodilation [27]–[30].

In contrast to the extensive studies that have been performed on HMGB1 in jawed vertebrates, little is known about the biological activities and physiological roles of HMGB1 in jawless lampreys. In the present study, we report for the first time the molecular cloning and characterization of an HMGB1 homolog from the Japanese lamprey (*L. japonica*). We examined Lj-HMGB1 expression in a number of tissues, but after treatment with LPS and ConA, we only found increased expression in the gills. Recombinant Lj-HMGB1 was revealed to bind to DNA and prevent DNA hydrolysis. In addition, the potential roles of Lj-HMGB1 in inflammation and cell proliferation were also investigated.

## Results

### Lamprey HMGB1 shares high sequence identities with other HMGB1/2/3 genes

**Figure 1:**
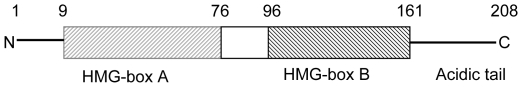
Schematic of the Lj-HMGB1 structural domains.

**Figure 2:**
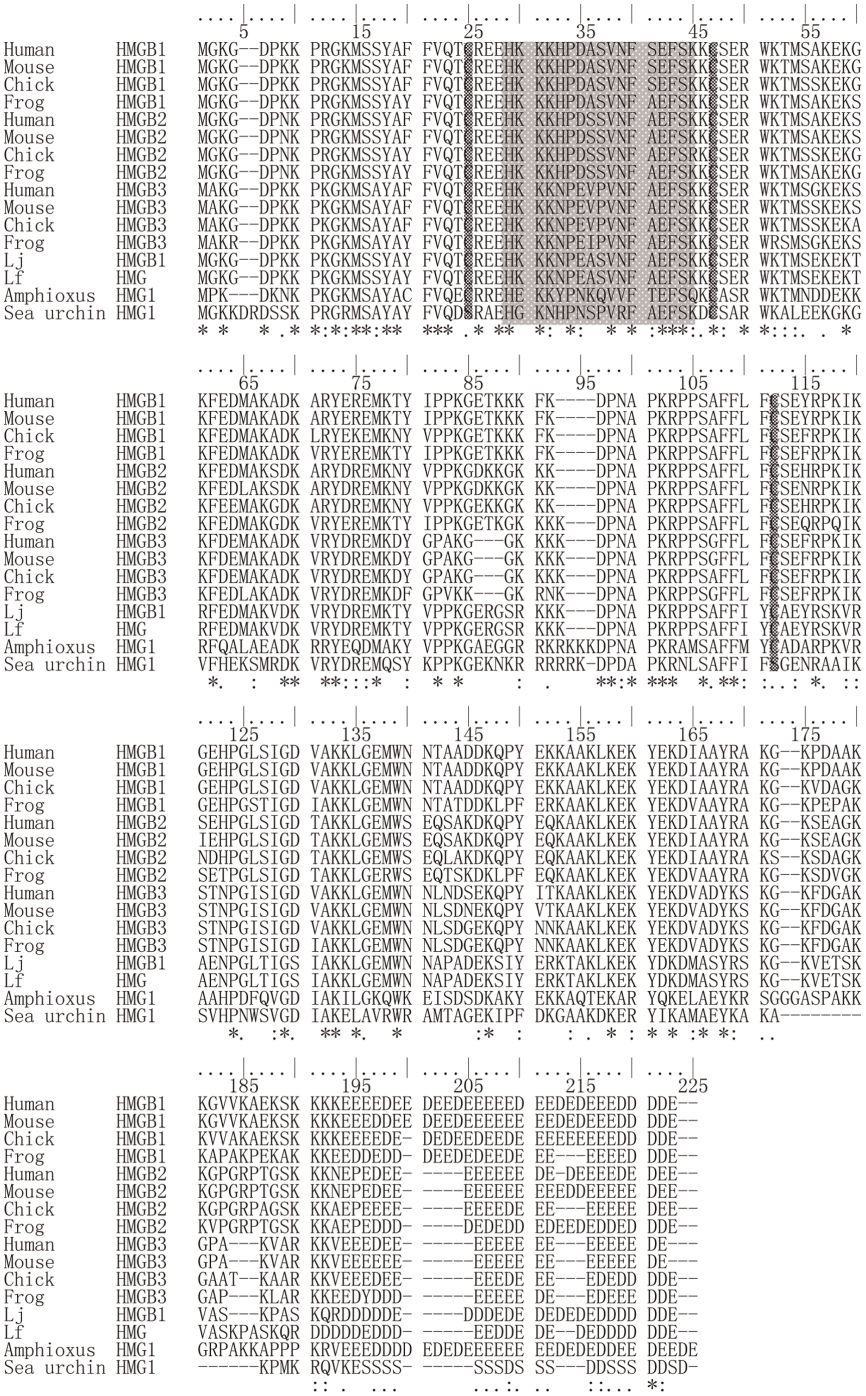
Sequence alignment of Lj-HMGB1 with HMGB1/2/3 of other species using ClustalX. The accession numbers of the amino acid sequences extracted from the EXPASY database are as follows: human HMGB1 (P09429); mouse HMGB1 (P63158); chick HMGB1 (Q9PUK9); frog HMGB1 (Q7SZ42); human HMGB2 (P26583); mouse HMGB2 (P30681); chick HMGB2 (P26584); frog HMGB2 (Q32NS7); human HMGB3 (O15347); mouse HMGB3 (O54879); chick HMGB3 (P40618); frog HMGB3 (Q1XCD9); Lj-HMGB1 (*Lampetra japonica*, HQ615991); Lf-HMGB1 (*Lampetra fluviatilis*, Q91070); amphioxus HMG1/2 (Q6PUE4); and sea urchin HMG1 (P40644). Identical (*asterisk*) and similar (*colon*) residues are indicated. *Dashes* represent gaps inserted into the alignment.

A full-length (1044-bp) cDNA of Lj-HMGB1 was identified from a cDNA library of lymphocyte-like cells in the Japanese lamprey. The cDNA included a 72-bp 5′-untranslated region (UTR) and a 345-bp 3′-UTR with a polyadenylation signal (AATAAA) at position 11 upstream of the poly (A) tail. The open reading frame (627 bp) encodes a polypeptide of 208 amino acids with an estimated molecular mass of 24,100 Da and a theoretical isoelectric point of 5.82 (ProtParam program of ExPASy, http://www.expasy.ch/tools/protparam.html). Similar to other HMGB1 proteins, Lj-HMGB1 contains an N-terminal HMG-box A domain (Pro_9_-Lys_76_), a central HMG-box B domain (Pro_96_-Ala_161_), a C-terminal acidic tail (Asp_184_-Glu_208_), and a linker (Thr_77_-Ala_95_) (Fig. 1). In addition, Lj-HMGB1 contains a positively charged amino acid sequence segment (His_27_-Lys_43_) and three cysteines (Cys_23_, Cys_45_, and Cys_107_) (Fig. 2). The nucleotide sequence of Lj-HMGB1 was submitted to the GenBank database with the accession number HQ615991.

**Figure 3:**
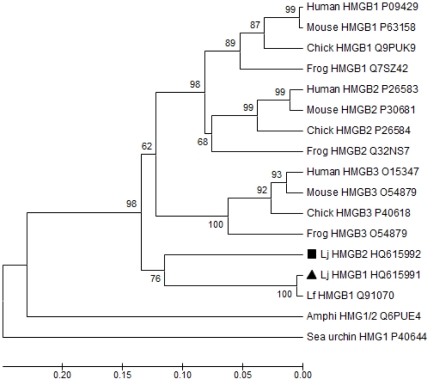
Phylogenetic relationship of lamprey HMGB1 with other HMGB members. A phylogenetic tree was constructed based on the amino acid sequences of HMGB from Fig. 2. The number at each node indicates the percentage of bootstrapping after 1000 replications. The bar (0.05) indicates genetic distance.

Multiple sequence alignments of Lj-HMGB1 with other HMGB genes revealed that it has 62.5–72.5% sequence homology with HMGB1/2/3 of other vertebrates (Fig. 2). Among the lampreys, Lj-HMGB1 differs from LfHMG1, another HMGB1 homolog that was identified from the larva of *Lampetra fluviatilis*, by only two amino acids [31]. Phylogenetic analysis indicated that Lj-HMGB1 should be placed outside of the vertebrate clade and that it represents the most divergent HMGB subfamily protein from mammals. In addition, Lj-HMGB1 is more closely related to vertebrate HMGBs than to the single HMG1 protein found in amphioxus and sea urchins (Fig. 3).

**Figure 4:**
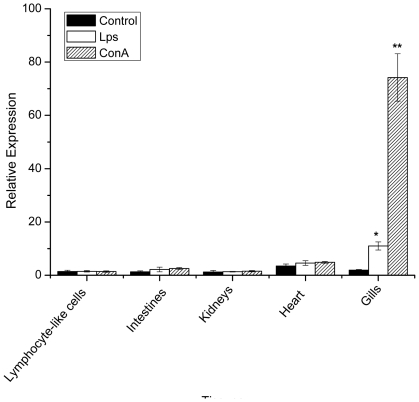
Lj-HMGB1 mRNA expression is significantly upregulated in gill tissue after treatment with LPS or ConA. The lamprey HMGB1 mRNA levels were determined using real-time quantitative RT-PCR in various lamprey tissues. Total RNA was extracted from the gills, intestines, heart, lymphocyte-like cells and kidneys of lampreys after stimulation with LPS or ConA. Lamprey GAPDH served as an internal control to calibrate the cDNA template for all of the samples, and PBS served as a negative treatment control. The significant differences (p<0.05) in HMGB1 expression between the challenged groups and the control group are indicated with asterisks.

To determine the expression pattern of Lj-HMGB1 in various tissues, we performed real-time quantitative PCR with total RNA extracted from lymphocyte-like cells and the kidneys, heart, gills, and intestines of lampreys after being challenged with LPS or ConA. Lj-HMGB1 was expressed slightly higher in heart tissue when treated with PBS (negative control), but only Lj-HMGB1 expression in the gills was significantly increased (5–30 fold) at 24 h after injecting either LPS or ConA (Fig. 4).

### Lamprey HMGB1 binds to polynucleotides and prevents DNA hydrolysis

**Figure 5:**
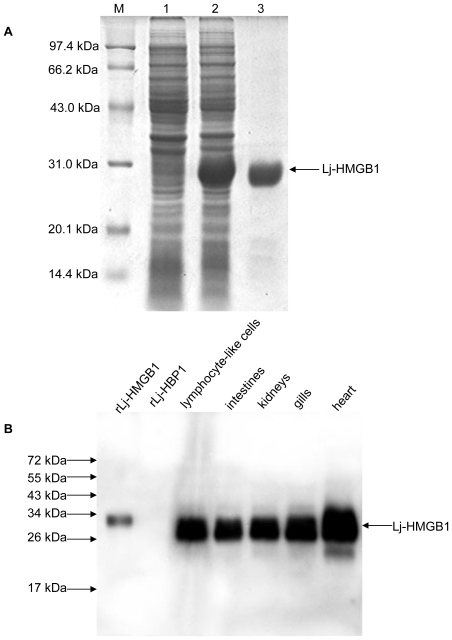
Expression and purification of recombinant Lj-HMGB1. A. SDS-PAGE. Total protein was separated by 12% SDS-PAGE under reducing conditions and stained with Coomassie Brilliant Blue R-250. Lane M, low molecular weight protein maker; Lane 1, crude lysate pre-induction; Lane 2, crude lysate post-induction; Lane 3, purified rLj-HMGB1. B. Western blot showing the specificity of the anti-Lj-HMGB1 antibody. Purified rLj-HMGB1 and proteins from crude homogenates of various lamprey tissues were probed with a rabbit anti-Lj-HMGB1 antibody. Lane 1, purified rLj-HMGB1; Lane 2, purified rLj-HBP1 (His-tagged negative control); Lanes 3–6, lymphocyte-like cells (lane 3), crude homogenate of the intestines (lane 4), kidneys (lane 5), gills (lane 6) and heart (lane 7) from *L. japonica*.

Recombinant lamprey HMGB1 protein (rLj-HMGB1) was expressed as a histidine-tagged fusion protein in *Rosetta blue* cells. The purified rLj-HMGB1 migrated as a single band on a 12% SDS-PAGE gel with a molecular mass of approximately 27,000 Da (Fig. 5A, lane 3). We next generated a rabbit anti-Lj-HMGB1 polyclonal antibody. Western blots showed that the anti-Lj-HMGB1 antibody recognized native Lj-HMGB1 in lamprey tissues, including the heart, kidneys, gills, intestines and lymphocyte-like cells. In addition, we noted that the concentration of Lj-HMGB1 protein in the heart tissue was slightly higher than in the other tissues examined, which was consistent with our real-time PCR results (Fig. 5B).

**Figure 6:**
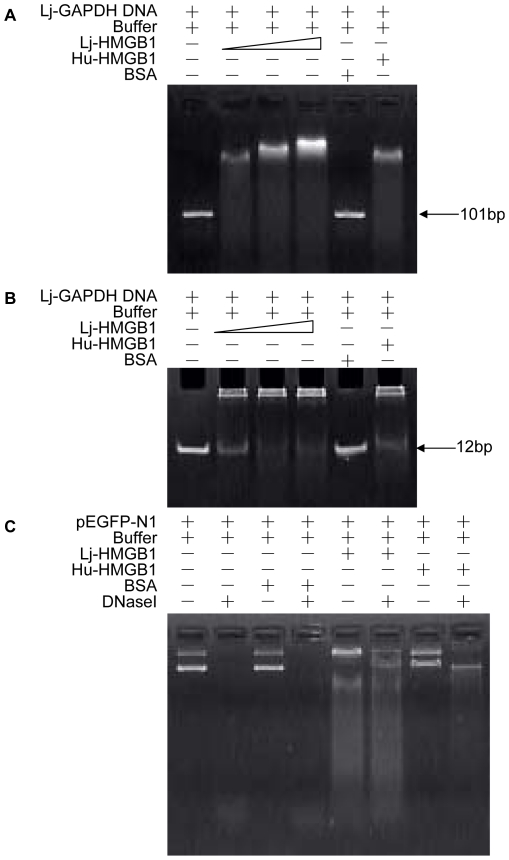
EMSA of lamprey HMGB1 binding to double-stranded polynucleotides. Double-stranded lamprey GAPDH DNA (100 ng, 101 bp and 12 bp in length) was incubated with purified Lj-HMGB1 protein at various concentrations, and aliquots were taken for electrophoresis on a 2% agarose gel (A) and a 20% native PAGE gel (B). Lane 1, Tris buffer; Lane 2, 10 ng of Lj-HMGB1; Lane 3, 20 ng of Lj-HMGB1; Lane 4, 30 ng of Lj-HMGB1; Lane 5, 30 ng of BSA; Lane 6, 10 ng of human HMGB1. (C). DNA hydrolysis in the presence of rLj-HMGB1. pEGFP-N1 DNA (4730 bp, 100 ng) was hydrolyzed by DNase I in the presence of rLj-HMGB1. Tris buffer (lane 2), BSA (lane 4), rLj-HMGB1 (lane 6) or human HMGB1 (lane 8) were incubated with pEGFP-N1 DNA at a quantitative ratio of 1∶10 in 20 mM Tris-HCl buffer containing 2 mM MgCl_2_ (pH 8.0) at room temperature for 10 min. DNase I (0.05 units, TaKaRa) was then added to each sample to hydrolyze pEGFP-N1 DNA at 37 °C for 10 min. Tris buffer (lane 1), BSA (lane 3), rLj-HMGB1 (lane 5) or human HMGB1 (lane 7) incubated with pEGFP-N1 DNA without DNase I served as the controls. The aliquots were analyzed on 1% agarose gels.

To investigate the DNA-binding ability of lamprey HMGB1, purified rLj-HMGB1 was incubated with lamprey glyceraldehyde-3-phosphate dehydrogenase (GAPDH) polynucleotides (101 bp and 12 bp in length), and the DNA-rLj-HMGB1 complexes were analyzed using electrophoretic mobility shift assays (EMSAs). We used human HMGB1 (Hu-HMGB1) as a positive control and observed the expected reduction in mobility with both the 101-bp and the 12-bp DNA fragments. In addition, the rLj-HMGB1 protein also reduced the mobility of the 101-bp and the 12-bp DNA bands in a dose-dependent manner (Fig. 6A, B), suggesting that the amount of rLj-HMGB1 limits the reaction. No reduction in DNA mobility was observed in the absence of rLj-HMGB1 or in the presence of BSA.

To confirm that Lj-HMGB1 binds directly to DNA, pEGFP-N1 DNA was incubated with rLj-HMGB1, BSA, or Hu-HMGB1 in the presence or absence of DNase I, as indicated in Fig. 6C. Both rLj-HMGB1 and Hu-HMGB1 protected the DNA from degradation, whereas BSA provided no protection, indicating that rLj-HMGB1 competitively binds to the pEGFP-N1 DNA and protects it from degradation.

### Lamprey HMGB1 stimulates TNF-α production in THP-1 monocytic cells and induces tumor cell proliferation

**Figure 7:**
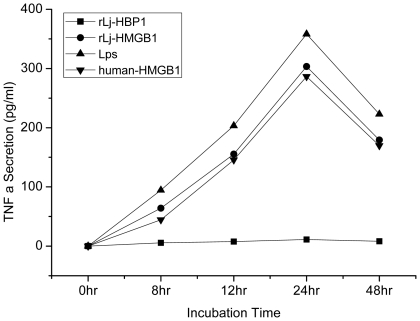
Lamprey HMGB1 induced TNF-α production in THP-1 monocytic cells. THP-1 cells (1×10^9^ cell/L) were incubated with rLj-HBP1 (50 ng/ml), rLj-HMGB1 (50 ng/ml), LPS (50 ng/ml), or human HMGB1 (50 ng/ml) for various lengths of time. Human HMGB1 was used as a positive control, and rLj-HBP1 was used as a negative control. The concentration of released TNF-α in the culture supernatants was measured by ELISA.

Mammalian HMGB1 has been shown to stimulate proinflammatory cytokines in monocytes [10]. We therefore sought to evaluate whether this evolutionarily divergent protein had a similar effect in monocytes. We measured the concentration of TNF-α released by human acute monocytic leukemia cells (THP-1) in the presence of rLj-HMGB1. rLj-HMGB1 (50 ng/ml) significantly stimulated the secretion of TNF-α from the THP-1 cells in a time-dependent manner. The highest TNF-α level (3.5 times higher) was detected after 24 h of incubation. The TNF-α level decreased thereafter and reached 50% of the maximum at 48 h. We compared the amount of TNF-α released in the presence of Lj-HMGB1 to that released when the cells were exposed to Hu-HMGB1 or LPS and found that similar amounts of TNF-α were released under all conditions. To ensure that contaminants from the purification process were not responsible for the release of TNF-α, we tested a related, but irrelevant, His-tagged protein (Lj-HBP1). Treatment with Lj-HBP1 did not cause a release of TNF-α (Fig. 7). Our results suggest that rLj-HMGB1 stimulates THP-1 monocytic cells to release proinflammatory cytokines.

**Figure 8:**
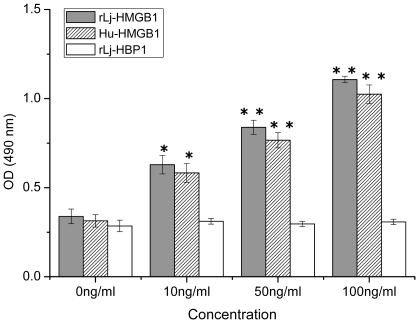
Lamprey HMGB1 induced proliferation of MCF-7 cells. MCF-7 cells were treated with different concentrations of rLj-HMGB1, Hu-HMGB1, or rLj-HBP1 for 24 h, and MTT assays were used to examine proliferation. Cells incubated with rLj-HBP1 were used as a negative control. “*" indicates a significant increase at p<0.05, “**" indicates a significant increase at p<0.01.

To determine whether rLj-HMGB1 can induce the proliferation of tumor cells, human breast adenocarcinoma cells (MCF-7) were cultured in the presence of rLj-HMGB1 (10–100 ng/ml) for 24 h. Compared with the negative control group (Lj-HBP1), rLj-HMGB1 and Hu-HMGB1 exhibited similar, significant increases in MCF-7 cell proliferation in a dose-dependent manner despite their evolutionary divergence (Fig. 8, p<0.05).

## Discussion

Lamprey HMGB1 shares over 70% sequence homology with human HMGB1, HMGB2 and HMGB3. Phylogenetic analysis using both a Poisson correction model and a P-distance model revealed that Lj-HMGB1 does not group with the HMGB1 subfamily phylogenetically (Fig. 3 and Appendix S1) when HMGB1 genes from sea urchin and amphioxus were used as the outgroup, respectively, suggesting that Lj-HMGB1 is divergent from the mammalian HMGB1 subfamily with regard to sequence identity. Coincident with our data, Sharman et al. hypothesized that LfHMG1 in *L. fluviatilis* diverged from the mammalian HMGB genes before mammalian HMGB split into the HMGB1 and HMGB2 subfamilies [31]. We documented 75% sequence identity between Lj-HMGB1 and Hu-HMGB1, which was insufficient to allow Lj-HMGB1 to group with the HMGB1 subfamily phylogenetically. However, we revealed that Lj-HMGB1 had a similar degree of effect to Hu-HMGB1 on TNF-α release and cell proliferation in human cell lines, suggesting that, despite not grouping together based on sequence, the two proteins are functionally similar.

Yang et al. reported that cysteine 106 in human HMGB1 in the cytokine-stimulating B box region is critical for the TLR4-dependent activation of macrophage TNF-α release [32]. Lj-HMGB1, an HMGB1 homolog, contains three cysteines, including one (Cys_107_) that is located at a position corresponding to the cysteine in human HMGB1, indicating that Lj-HMGB1 may act as a cytokine in lampreys. The structural similarity between lamprey and human HMGB1 suggest that Lj-HMGB1 may also play a role in multiple biological functions that are important for survival. The highly conserved HMGB1 of jawed vertebrates is involved in many crucial biological functions in cells, such as differentiation, cytoskeletal reorganization, migration, apoptosis, and proliferation [33]–[35].

LPS and ConA have been commonly used in lamprey, fish and amphioxus immunological studies [36]–[38]. LPS is the major component of the outer membrane of Gram-negative bacteria. In mammals, TLR4 is the central component of the LPS receptor [39]. In response to LPS, there is a delayed release of HMGB1 from jawed vertebrate monocytes or macrophages, which in turn induces the production of proinflammatory mediators, such as TNF-α and IL-1β [40]. ConA is a plant mitogen and is known for its ability to stimulate mouse T-cell subsets [41]. ConA can also initiate cell division (mitogenesis) by rapidly stimulating the metabolism of thymocytes to produce T-lymphocytes [42]. After challenging lampreys with LPS or ConA, a significant up-regulation of Lj-HMGB1 expression was observed in the gills (Fig. 4) but not elsewhere in the organism. The fact that LPS/ConA up-regulated Lj-HMGB1 expression suggests that Lj-HMGB1 may trigger an inflammatory response in lamprey gills. Based on the fact that lampreys have thymus-like lympho-epithelial structures in the tips of the gill filaments and the neighboring secondary lamellae [43], the injection of LPS and ConA may activate B- and T-like lymphocytes in the gills to release proinflammatory cytokines, such as Lj-HMGB1, that participate in the immune response.

Andersson et al. revealed HMGB1 modulates the inflammatory cascade in LPS-activated macrophages by inducing the production of the proinflammatory cytokines TNF-α and IL-1β [44]. Because there are no lamprey cell lines, we must speculate on the functions of Lj-HMGB1 using human cell lines. To this end, we determined that the lamprey HMGB1 protein induces the release of TNF-α from THP1 cells, indicating that Lj-HMGB1 is capable of regulating the mammalian innate immune system that defends animals from the invasion of pathogens and other exogenous injuries to a similar extent as Hu-HMGB1. Moreover, real-time quantitative PCR shows that LPS may stimulate increased production of Lj-HMGB1 in vivo, suggesting that LPS may also induce the release of additional HMGB1 from THP1 cells. This delayed release of HMGB1 from THP1 cells may, in turn, stimulate the generation of TNF-α and other proinflammatory cytokines (Fig. 7). Thus, Lj-HMGB1 may act as a key element in the signaling pathway that leads from LPS stimulation to TNF-α production. However, further studies are needed to clarify this question. Previous work has suggested that TLR14a and TLR14b genes are present in the lamprey [45]. Recently, we identified a Toll-like receptor 2/3 and a TNF-alpha receptor-associated factor in the sea lamprey genome database (http://asia.ensembl.org/Petromyzon_marinus/Info/Index), indicating that Lj-HMGB1 may also act as a cytokine in lampreys.

In addition to examining Lj-HMGB1's ability to bind to DNA, we investigated the ability of rLj-HMGB1 to protect DNA from degradation. Our results suggested that the rLj-HMGB1 protein can bind to DNA and protect it from degradation (Fig. 6A–C). DNA damage is known to be an extremely important event during cell dysfunction or apoptosis.

Sundberg et al. reported that exogenous HMGB1 acted as a proliferation signal for activated T lymphocytes [46]. In addition, Bassi et al. reported that exogenous HMGB1 affected the growth of human T98G glioblastoma cells [47]. To better understand the function of lamprey HMGB1, we investigated the effect of Lj-HMGB1 on MCF-7 cells. We found that Lj-HMGB1 also regulates the proliferation of MCF-7 cells, which leads us to speculate that lamprey HMGB1 may have similar biological functions to HMGB1 in jawed vertebrates and may be critical to the survival of lampreys.

**Figure 9:**
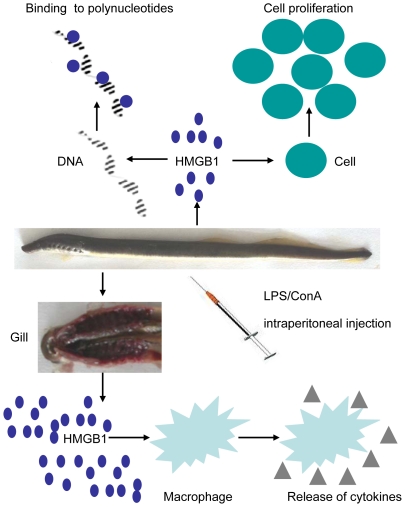
Model of the functions of lamprey HMGB1. Lamprey HMGB1 has various roles, such as binding DNA, which may maintain DNA structure, and inducing cell proliferation, which may promote the evolutional stability of the parasitic lamprey. After LPS/ConA stimulation, the Lj-HMGB1 gene was up-regulated in gills, which in turn promoted the release of proinflammatory mediators, such as TNF-α, to alert the lampreys to underlying danger and prevent the invasion of bacteria or viruses or other exogenous injuries.

Given these confirmed biological functions of Lj-HMGB1 in preventing cell apoptosis, promoting cell proliferation and mediating the immune response, we speculate that lamprey HMGB1 may have similar biological functions to HMGB1 in jawed vertebrates. The conserved function and sequence identity of HMGB1 suggest that the protein is likely critical for the survival of the animal and that HMGB1 of the jawed vertebrates may have originated from an ancient animal that lived 350 million years ago. The characterization of lamprey HMGB1 may have important consequences even in highly divergent species (Fig. 9).

## Materials and Methods

### Animals

The handling of lampreys and all experimental procedures were approved by the Animal Welfare and Research Ethics Committee of the Institute of Dalian Medical University (Permit Number: SYXK2004—0029). We performed the animal experiments at the Institute of Dalian Medical University. Adult lampreys (*L. japonica*) were obtained in December 2010 from the Tongjiang Valley of Songhua River, Heilongjiang Province, China. The animals (three in each group) were intraperitoneally injected with PBS, LPS (10 µg), or ConA (10 µg).

### Cloning of lamprey HMGB1

Construction of a cDNA library from lamprey lymphocyte-like cells and expressed sequence tags (EST) sequencing were previously completed by our lab. Based on EST analysis, a full-length lamprey HMGB1 homolog was identified using NCBI's Basic Local Alignment Search Tool (BLAST). Total RNA was isolated from lamprey lymphocyte-like cells using Trizol (Invitrogen, USA), and cDNA was synthesized with the High Fidelity PrimeScript™ RT-PCR Kit (TaKaRa, China). The PCR primers 5′-TGAGCAGCAAGTGTCGGTGA-3′ (forward) and 5′-GCCAAACCGTGAAGTAAGCC-3′ (reverse) were designed based on the EST most homologous to HMGB1. The PCR product was purified, cloned into a pMD19-T vector using a DNA Ligation kit (TaKaRa, China), and transformed into DH5α *E. coli*. DNA sequencing was conducted with M13 Forward/Reverse primers using a model ABI 377 DNA Sequencer (Applied Biosystems, USA).

### Sequence alignment and construction of a phylogenetic tree

A total of 16 HMGB1/2/3 sequences from other species were obtained from ExPASy (http://www.expasy.ch/tools/blast/, Appendix S1). Multiple sequence alignments were performed using ClustalX (http://www.ebi.ac.uk/Tools/clustalw/). For the construction of the phylogenetic tree, the program MEGA 4 was used. A neighbor-joining tree was constructed based on the pair-wise deletion of gaps/missing data and a p-distance matrix of an amino acid model with 1000 bootstrapped replicates [48].

### Real-time PCR

Total RNA was extracted from the lamprey tissues 24 hours post injection using Trizol (Invitrogen, USA), and the RNA was treated with DNase I (TaKaRa, China). Reverse transcription was performed as previously described [49]. Real-time quantitative PCR was performed with the TaKaRa SYBR® PrimeScript™ RT-PCR Kit according to the manufacturer's protocol. Each reaction contained 1×SYBR *Premix Ex* Taq, 10 µM of each primer, and 2 µl of cDNA (100 ng/µl) in a final volume of 25 µl. The amplification was performed in a TaKaRa PCR Thermal Cycler Dice Real Time System with the following parameters: initial denaturation at 95 °C for 10 s to activate the DNA polymerase, followed by 45 cycles of 5 s at 95 °C, 30 s at 60 °C, and 30 s at 72 °C. The Lj-HMGB1 specific primers were 5′-CCCGTCGGCTTTCTTCATC-3′ (forward) and 5′-TTCCACATCTCACCCAGTTTCTT-3′ (reverse). GAPDH (GenBank number AY578058) was included as an internal control. Each sample was analyzed in triplicate. The data were analyzed with the Thermal Cycler Dice Real Time System analysis software (TaKaRa, China).

### Cloning, expression, and purification of the lamprey HMGB1 protein

The open reading frame (ORF) of Lj-HMGB1, flanked by an *EcoR*I and a *Not*I restriction site, was amplified and subcloned into a pET 28a vector with a His-tag. The recombinant Lj-HMGB1 was expressed in *Rosetta blue* cells induced with 1 mM isopropyl-1-thio-β-D-galactopyranoside (IPTG) for 4 h. Subsequently, the cells were collected by centrifugation, washed, and resuspended in 20 mM Tris-HCl containing 1 mM EDTA (pH 8.0). The cell suspension was sonicated for 30 min on ice and centrifuged again at 14,000 rpm for 20 min at 4 °C. The soluble supernatant was collected and subjected to a Ni-NTA His-Bind resin column (Novagen, USA) equilibrated with binding buffer (20 mM Tris-HCl, pH 8.0, 150 mM NaCl, 10 mM imidazole). After washing the column with washing buffer (20 mM Tris-HCl, pH 8.0, 150 mM NaCl, 50 mM imidazole), the recombinant protein was collected in elution buffer containing 20 mM Tris-HCl (pH 8.0), 150 mM NaCl, 200 mM imidazole. The concentration of rLj-HMGB1 was measured using a Bicinchoninic Acid (BCA) Protein Assay kit (Beyotime, China). The purified rLj-HMGB1 was analyzed by 12% SDS-PAGE using the method of Laemmli [50] and stored at −80 °C.

### Preparation of anti-lamprey HMGB1 polyclonal antibody and Western blots

A polyclonal antibody against rLj-HMGB1 was generated by subcutaneous injection of the purified protein (2 mg, emulsified with Freund's adjuvant) into adult New Zealand White Rabbits as described previously [26]. After 6 subsequent, increasing immunizations at 1-week intervals, blood was drawn from a carotid artery, and the antibody was purified using Protein A MagBeads (GenScript, USA). The antibody titer was determined by enzyme-linked immunosorbent assay (ELISA). Total proteins were extracted from lamprey tissues using a cell lysis buffer (Beyotime, China), and the protein concentration was measured using the Bicinchoninic Acid (BCA) Protein Assay kit (Beyotime, China). For Western blots, 10 µg of purified rLj-HMGB1 and total protein from the different lamprey tissues were subjected to 12% SDS-PAGE and transferred onto nitrocellulose membranes. The membranes were blocked with 5% skimmed milk and incubated with rabbit anti-Lj-HMGB1 (1∶1000 dilution) antibody overnight at 4 °C followed by incubation with HRP-conjugated goat anti-rabbit IgG (1∶5000). The membrane was developed with the ECL substrate (Beyotime, China).

### TNF-α assay of human acute monocytic leukemia cells

To quantify the production of TNF-α from the Lj-HMGB1-activated macrophages, THP-1 monocytic cells purchased from American Type Culture Collection were cultured in RPMI-1640 medium (Invitrogen, Carlsbad, CA) containing 10% fetal bovine serum, 2 mM glutamine, and 1% streptomycin/penicillin. Then, 1×10^9^ cells/L were stimulated with an irrelevant His-tagged protein (50 ng/ml), rLj-HMGB1 (50 ng/ml), LPS (50 ng/ml), or human HMGB1 (50 ng/ml) (Sigma, USA). The culture supernatants were collected at 0, 8, 12, 24, and 48 h post-stimulation, and the TNF-α levels were measured by ELISA (Boster Biological Technology, China). The samples were assayed in duplicate, and each experiment was repeated at least three times.

### Electrophoretic mobility shift assay (EMSA)

Lamprey GAPDH DNAs (101 bp and 12 bp) were synthesized (TaKaRa, China). For effective annealing, one additional nucleotide was added at the 5′ terminus of each sequence. The polynucleotides were denatured at 95 °C for 5 min and annealed by cooling down slowly to room temperature. The concentration of the annealed dsDNA was determined based on the absorbance at 260 nm on a UV-1800 PC spectrophotometer. BSA (30 ng), human HMGB1 (10 ng) and rLj-HMGB1 (10 ng, 20 ng, or 30 ng) were incubated with 100 ng dsDNA in 10 µl of TNME solution (20 mM Tris buffer, pH 7.2, 50 mM NaCl, 0.5 mM DTT, 1 mM MgCl_2_, and 0.5 mM EDTA) for 20 min at room temperature. The DNA-protein complexes were electrophoresed on 2% agarose or 20% non-denaturing polyacrylamide gels. The gels were stained with ethidium bromide (0.5 µg/ml) for 30 min, and the DNA was visualized using the Imaging Digital Science Electrophoresis Documentation and Analysis System (EDAS) 290 (Eastman Kodak, New Haven, CT, USA).

### Hydrolysis of DNA with DNase I in the presence of rLj-HMGB1

rLj-HMGB1, BSA and human HMGB1 were incubated with pEGFP-N1 DNA (4730 bp, 100 ng) at a ratio of 1∶10 in 20 mM Tris-HCl containing 2 mM MgCl_2_ at a pH of 8.0 and at room temperature for 10 min. 0.05 units DNase I (TaKaRa, China) was added to the mixture to hydrolyze the DNA at 37 °C for 10 min, followed by the addition of 5 mM EDTA to stop the enzymatic reaction. The digested DNA was electrophoresed on 1% agarose gels. The pEGFP-N1 plasmid with DNA alone served as a negative control.

### Proliferation of human breast adenocarcinoma cells

Human breast adenocarcinoma cells (MCF-7) purchased from American Type Culture Collection were plated onto 96-well plates at a density of 1×10^4^ cells/well in 200 µl of RPMI-1640 media containing 10% fetal bovine serum, 2 mM glutamine, and 1% streptomycin/penicillin. After the cells were seeded on the plate with uniform attachment for at least one day, the cells were treated with 200 µl of fresh medium containing rLj-HMGB1, an irrelevant His-tagged protein or human HMGB1 (10, 50, and 100 ng/ml) for 24 h. After the medium was removed from the plates, the treated cells were incubated with MTT at a final concentration of 50 µg in the dark for 4 h followed by the addition of 100 µl DMSO. The plate was read with a microplate spectrophotometer at 490 nm. Cell viability was estimated using the colorimetric method based on the ability of the cellular dehydrogenases of viable cells to reduce 3-(4,5-dimethylthiazol-2-yl)-2,5-diphenyl tetrazolium bromide (MTT) from a yellow, water-soluble dye to a dark blue, insoluble formazan product. All MTT assays were performed four times.

### Statistical analysis

All data are presented as the means ± SE based on separate experiments. Student's t- tests were used to determine statistical significance.

## Supporting Information

Appendix S1 Amino acid sequences of HMGB1/2/3 of lamprey and other species and phylogenetic analysis using both a Poisson correction model and a P-distance model and different methods, such as UPGMA, Fitch-Margoliash.
(DOC)
