# Supplementary material for: Identification and Characterization of the Lamprey High-Mobility Group Box 1 Gene
Source: PLoS One. 2012 Apr 26;7(4):e35755. doi: 10.1371/journal.pone.0035755 (PMC3338530; doi:10.1371/journal.pone.0035755)
Supplement: Appendix S1 — Amino acid sequences of HMGB1/2/3 of lamprey and other species and phylogenetic analysis using both a Poisson correction model and a P-distance model and different methods, such as UPGMA, Fitch-Margoliash. (DOC) [file pone.0035755.s001.doc]

Appendix S1 Legends

>Human_HMGB1_P09429

MGKGDPKKPRGKMSSYAFFVQTCREEHKKKHPDASVNFSEFSKKCSERWKTMSAKEKGKFEDMAKADKARYEREMKTYIPPKGETKKKFKDPNAPKRPPSAFFLFCSEYRPKIKGEHPGLSIGDVAKKLGEMWNNTAADDKQPYEKKAAKLKEKYEKDIAAYRAKGKPDAAKKGVVKAEKSKKKKEEEEDEEDEEDEEEEEDEEDEDEEE DDDDE

>Mouse_HMGB1_P63158

MGKGDPKKPRGKMSSYAFFVQTCREEHKKKHPDASVNFSEFSKKCSERWKTMSAKEKGKFEDMAKADKARYEREMKTYIPPKGETKKKFKDPNAPKRPPSAFFLFCSEYRPKIKGEHPGLSIGDVAKKLGEMWNNTAADDKQPYEKKAAKLKEKYEKDIAAYRAKGKPDAAKKGVVKAEKSKKKKEEEDDEEDEEDEEEEEEEEDEDEEEDDDDE

>Chick_HMGB1_Q9PUK9

MGKGDPKKPRGKMSSYAFFVQTCREEHKKKHPDASVNFSEFSKKCSERWKTMSSKEKGKFEDMAKADKLRYEKEMKNYVPPKGETKKKFKDPNAPKRPPSAFFLFCSEFRPKIKGEHPGLSIGDVAKKLGEMWNNTAADDKQPYEKKAAKLKEKYEKDIAAYRAKGKVDAGKKVVAKAEKSKKKKEEEEDEDEDEEDEEDEEEEEEEEEDDDDE

>Frog_HMGB1_Q7SZ42

MGKGDPKKPRGKMSSYAYFVQTCREEHKKKHPDASVNFAEFSKKCSERWKTMSAKEKSKFEDMAKADKVRYEREMKTYIPPKGETKKKFKDPNAPKRPPSAFFLFCSEFRPKIKGEHPGSTIGDIAKKLGEMWNNTATDDKLPFERKAAKLKEKYEKDVAAYRAKGKPEPAKKAPAKPEKAKKKEEDDEDDDEEDEDEEDEEEEEEEDDDE

>Human_HMGB2_P26583

MGKGDPNKPRGKMSSYAFFVQTCREEHKKKHPDSSVNFAEFSKKCSERWKTMSAKEKSKFEDMAKSDKARYDREMKNYVPPKGDKKGKKKDPNAPKRPPSAFFLFCSEHRPKIKSEHPGLSIGDTAKKLGEMWSEQSAKDKQPYEQKAAKLKEKYEKDIAAYRAKGKSEAGKKGPGRPTGSKKKNEPEDEEEEEEEEDEDEEEEDEDEE

>Mouse_HMGB2_P30681

MGKGDPNKPRGKMSSYAFFVQTCREEHKKKHPDSSVNFAEFSKKCSERWKTMSAKEKSKFEDLAKSDKARYDREMKNYVPPKGDKKGKKKDPNAPKRPPSAFFLFCSENRPKIKIEHPGLSIGDTAKKLGEMWSEQSAKDKQPYEQKAAKLKEKYEKDIAAYRAKGKSEAGKKGPGRPTGSKKKNEPEDEEEEEEEEEEEDDEEEEEDEE

>Chick_HMGB2_P26584

MGKGDPNKPRGKMSSYAYFVQTCREEHKKKHPDSSVNFAEFSRKCSERWKTMSSKEKGKFEEMAKGDKARYDREMKNYVPPKGEKKGKKKDPNAPKRPPSAFFLFCSEHRPKIKNDHPGLSIGDTAKKLGEMWSEQLAKDKQPYEQKAAKLKEKYEKDIAAYRAKSKSDAGKKGPGRPAGSKKKAEPEEEEEEEEDEEEEEEEEDEE

>Frog_HMGB2_Q32NS7

MGKGDPNKPRGKMSSYAYFVQTCREEHKKKHPDSSVNFAEFSKKCSERWKTMSAKEKGKFEDMAKGDKVRYEREMKTYIPPKGETKGKKKKDPNAPKRPPSAFFLFCSEQRPQIKSETPGLSIGDTAKKLGERWSEQTSKDKLPFEQKAAKLKEKYEKDVAAYRAKGKSDVGKKVPGRPTGSKKKAEPEDDDDEDEDDEDEEDEDDEDDDE

>Human_HMGB3_O15347

MAKGDPKKPKGKMSAYAFFVQTCREEHKKKNPEVPVNFAEFSKKCSERWKTMSGKEKSKFDEMAKADKVRYDREMKDYGPAKGGKKKKDPNAPKRPPSGFFLFCSEFRPKIKSTNPGISIGDVAKKLGEMWNNLNDSEKQPYITKAAKLKEKYEKDVADYKSKGKFDGAKGPAKVARKKVEEEDEEEEEEEEEEEEEEDE

>Mouse_HMGB3_O54879

MAKGDPKKPKGKMSAYAFFVQTCREEHKKKNPEVPVNFAEFSKKCSERWKTMSSKEKSKFDEMAKADKVRYDREMKDYGPAKGGKKKKDPNAPKRPPSGFFLFCSEFRPKIKSTNPGISIGDVAKKLGEMWNNLSDNEKQPYVTKAAKLKEKYEKDVADYKSKGKFDGAKGPAKVARKKVEEEEEEEEEEEEEEEEEEDE

>Chick_HMGB3_P40618

MAKGDPKKPKGKMSAYAFFVQTCREEHKKKNPEVPVNFAEFSKKCSERWKTMSSKEKAKFDEMAKADKVRYDREMKDYGPAKGGKKKKDPNAPKRPPSAFFLFCSEFRPKIKSTNPGISIGDVAKKLGEMWNNLSDGEKQPYNNKAAKLKEKYEKDVADYKSKGKFDGAKGAATKAARKKVEEEDEEEEE DEEEEDEDDDDE

>Frog_HMGB3_O54879

MAKRDPKKPKGKMSAYAYFVQTCREEHKKKNPEIPVNFAEFSKKCSERWRSMSGKEKSKFEDLAKADKVRYDREMKDFGPVKKGKRNKDPNAPKRPPSGFFLFCSEFRPKIKSTNPGISIGDIAKKLGEMWNNLSDGEKQPYNNKAAKLKEKYEKDVADY KSKGKFDGAKGAPKLARKKEEDYDDDEEEEEDEEDEEEDDE

>Lj_HMGB1_HQ615991

MGKGDPKKPKGKMSSYAYFVQTCREEHKKKNPEASVNFAEFSKKCSERWKTMSEKEKTRFEDMAKVDKVRYDREMKTYVPPKGERGSRKKKDPNAPKRPPSAFFIYCAEYRSKVRAENPGLTIGSIAKKLGEMWNNAPADEKSIYERKTAKLKEKYDKDMASYRSKGKVETSKVASKPASKQRDDDDDEDDDEDEDEDEDEDDDDDDE

>Lf_HMGB1_Q91070

MGKGDPKKPKGKMSSYAYFVQTCREEHKKKNPEASVNFAEFSKKCSERWKTMSEKEKTRFEDMAKVDKVRYDREMKTYVPPKGERGSRKKKDPNAPKRPPSA

FFIYCAEYRSKVRAENPGLTIGSIAKKLGEMWNNAPADEKSIYERKTAKLKEKYDKDMASYRSKGKVETSKVASKPASKQRDDDDDEDDDEEDDEDEDEDDDDDDE

>Lj_HMGB2_HQ615992

MGKGEPGKPRGKMSSYAYFVQTCREEHKKKHPEASVNFAEFSKKCSERWKTMSPKEKARFEEMAKADKARYDREMKNYVPPKGKRKTKDPNAPKRPPSAFFVFCSEHRPKVKADHPGLGIGEIAKRLGEMWGLLTPETKSPYEKKAAKLKEKYEKDVAAYRGKGKAAPSKAAPEEDDDEEDEEEEDDDDEDDDE

>Amphi_HMG1/2_Q6PUE4

MPKDKNKPKGKMSAYACFVQECRREHEKKYPNKQVVFTEFSQKCASRWKT MNDDEKKRFQALAEADKRRYEQDMAKYVPPKGAEGGRRKRKKKDPNAPKRAMSAFFMYCADARPKVRAAHPDFQVGDIAKILGKQWKEIS DSDKAKYEKK AQTEKARYQKELAEYKRSGGGASPAKKGRPAKKAPPPKRVEEEDDDDEDEDEEEEEEEED EDEDDEEDEE DE

>Sea_urchin_HMG1_P40644

MGKKDRDSSKPRGRMSAYAYFVQDSRAEHGKNHPNSPVRFAEFSKDCSARWKALEEKGKGVFHEKSMRDKVRYDREMQSYKPPKGEKNKRRRRRKDPDAP KRNLSAFFIFSGENRAAIKSVHPNWSVGDIAKELAVRWRAMTAGEKIPFDKGAAKDKERY IKAMAEYKAK AKPMKRQVKE SSSSSSSDSS SDDSSSDDSD

Phylogenetic analysis using both a Poisson correction model and a P-distance model. In addition, phylogenetic trees constructed using other methods, such as UPGMA, Fitch-Margoliash.

Model: p-distance; outgroup: Amphi; method: N-J

Model: Poisson correction; outgroup: Amphi; method: N-J

Model: p-distance; outgroup: Amphi; method: N-J

Model: Poisson correction; outgroup: Amphi; method: N-J

Model: p-distance; outgroup: sea urchin; method: N-J

Model: Poisson correction; outgroup: sea urchin; method: N-J

Model: Poisson correction; outgroup: sea urchin; method: minimum evolution, ME)

Model: Poisson correction; outgroup: sea urchin; method: unweighted-pair-group method with arithmetic means, UPGMA)
